# Near-Surface Material Phases and Microstructure of Scandate Cathodes

**DOI:** 10.3390/ma12040636

**Published:** 2019-02-20

**Authors:** Xiaotao Liu, Bernard K. Vancil, Matthew J. Beck, Thomas John Balk

**Affiliations:** 1 Department of Chemical and Materials Engineering, University of Kentucky, 177 F. Paul Anderson Tower, Lexington, KY 40506, USA; xli323@g.uky.edu (X.L.); beck@engr.uky.edu (M.J.B.); 2 E Beam, Inc., 14925 SW Barrows Road, Beaverton, OR 97007, USA; bernie@ebeaminc.com

**Keywords:** scandate cathodes, elemental mapping, electron diffraction, 3D tomography, 3D reconstruction

## Abstract

Scandate cathodes that were fabricated using the liquid-solid process and that exhibited excellent emission performance were characterized using complementary state-of-the-art electron microscopy techniques. Sub-micron BaAl_2_O_4_ particles were observed on the surfaces and edges of tungsten particles, as seen in cross-section samples extracted from the scandate cathode surface regions. Although several BaAl_2_O_4_ particles were observed to surround smaller Sc_2_O_3_ nanoparticles, no chemical mixing of the two oxides was detected, and in fact the distinct oxide phases were separately verified by chemical analysis and also by 3D elemental tomography. Nanobeam electron diffraction confirmed that the crystal structure throughout W grains is body-centered cubic, indicating that they are metallic W and did not experience noticeable changes, even near the grain surfaces, as a result of the numerous complex chemical reactions that occur during cathode impregnation and activation. 3D reconstruction further revealed that internal Sc/Sc_2_O_3_ particles tend to exhibit a degree of correlated arrangement within a given W particle, rather than being distributed uniformly throughout. Moreover, the formation of Sc/Sc_2_O_3_ particles within W grains may arise from W surface roughening that occurs during the liquid-solid synthesis process.

## 1. Introduction

Scandia-doped tungsten thermionic cathodes (referred to as scandate cathodes) are promising candidates for application in microwave tubes [1,2], traveling wave tubes (TWTs) [3,4], satellite communication [5] and vacuum electron devices (VEDs) [6,7,8,9,10] owing to their reported enhancement of electron emission, delivering higher current densities at lower temperatures [1,3,5,6,8,11,12] than conventional dispenser cathodes, i.e., oxide cathodes [13,14,15,16], B-type cathodes [17,18,19,20], and M-type cathodes [21,22,23]. Consequently, scandate cathode technology has received significant attention over recent decades. Various scandate cathodes have been developed, including impregnated [24,25,26], pressed [27,28], and top-layered types [29,30,31]. Of these, scandate cathodes fabricated from starting powders of micron-scale tungsten (W) and nanoscale scandia (Sc_2_O_3_) are reported to exhibit the most promising emission characteristics and have been widely investigated. This scandate cathode variant is a powder metallurgy (P/M) porous tungsten plug fabricated with nanosized scandia-doped tungsten powder and impregnated with barium calcium aluminate (in a specific molar ratio) prior to activation at the proper temperature. In order to enable commercialization and application of scandate cathodes, however, several issues must first be addressed, including emission uniformity, poor reproducibility, and an incomplete understanding of the mechanisms that govern emission [7,16,19,27,32,33,34,35].

These persistent issues are due, at least to some extent, to knowledge gaps in the relationships between processing, microstructure, composition, and emission properties of scandate cathodes. Unlike simple materials such as a binary alloy or binary oxide system, impregnated scandate cathodes contain various multi-element compounds, scandia-doped W powder mixes, and impregnate materials. In addition, numerous complex reactions occur during the fabrication steps, principally during impregnation and activation processes [27,34]. These issues lead to exceptional difficulty in understanding exactly which phases are present at key locations in scandate cathodes, and which phases are most important to electron emission at service temperature. Apart from the complexity of the cathode microstructure itself, inherent limits to characterization techniques have hindered scandate cathode studies, even though the first scandate cathode was produced in 1967 [36]. Fortunately, instrumentation for materials research, especially electron microscopy, has recently undergone rapid and substantial development, facilitating more advanced investigations of scandate cathodes and an improved understanding of their relevant nanoscale features.

Recent studies by the current authors [6,37] indicate that the surfaces of scandate cathodes that emit well also exhibit highly faceted W grains decorated with nanoscale Sc_2_O_3_, BaAl_2_O_4_ and Ba/BaO particles. Importantly, even though a ~100 nm thick surface layer containing Ba–Sc–O has been invoked in certain emission models [7,32], no such surface layer or region was detected at or near the cathode surfaces in this study. During the advanced characterization experiments described here, primary focus was on nanoscale structure and composition within the near-surface regions, to better understand the cathode features thought to be involved in thermionic electron emission. This work has yielded new insights into the structure and composition of phases that are revealed in cross-section specimens from scandate cathode surface regions, and which provide a basis for modeling improved emission performance.

## 2. Materials and Methods

The scandate cathodes characterized in this paper were prepared with W precursor powder mixed with nanoscale scandia, and this powder mixture had been prepared using the liquid-solid (L-S) technique. Detailed procedures of the L-S approach can be found in an earlier paper by one of the authors [1]. The L-S process utilizes an aqueous nitrate solution to dissolve scandia, and then solid tungsten powder is added as a substrate for formation of scandia nanoparticles. The nitrate solution may etch or roughen the surfaces of tungsten particles. Multiple scandate cathodes were provided by e beam inc., where emission testing was also performed. Fabrication included the following steps: for each cathode, the W-scandia powder mixture was pressed into a pellet ~2 mm diameter and ~1 mm tall; then the pellet was sintered and subsequently impregnated with barium calcium aluminate (6BaO–1CaO–2Al_2_O_3_); afterwards, the sample was washed with deionized water to remove residual impregnate material; finally, the cathodes were activated by heating at 1150 °C_b_ for 1 h. Prior to microstructural characterization, two cathodes (designated cathode #1 and cathode #2) were emission tested in a close-spaced diode (CSD) configuration. These scandate cathodes exhibited excellent emission capabilities as indicated by knee temperatures of 820 °C_b_ and 837 °C_b_ (brightness temperature, with respect to W) at the end of extensive emission testing: 3024 and 9840 h, respectively.

Morphological characterization and location-specific chemical analysis of scandate cathode surfaces was performed in a dual-beam focused ion beam and scanning electron microscope (FIB-SEM, FEI Helios NanoLab 660, Thermo Fisher Scientific, Hillsboro, OR, USA) equipped with an X-ray energy dispersive spectrometer (EDS; Oxford X-Max 80 mm^2^ detector, Oxford Instruments, Abingdon, UK). Two lift-out lamellae were extracted from cathode #1 using the FIB and were examined in a transmission electron microscope (TEM; FEI Talos F200X (Thermo Fisher Scientific, Hillsboro, OR, USA) and JEOL JEM-F200 microscopes (JEOL, Tokyo, Japan) were used in this study). Along with scanning transmission electron microscope (STEM) imaging for structural characterization and scanning nanobeam electron diffraction for crystal structure determination, high-resolution elemental mapping was performed on the TEM cross-section lamella using the Super-X EDS system (which includes 4 confocal EDS detectors that simultaneously collect X-rays from the same sample location) in the FEI Talos F200X TEM. Serial sectioning for 3D reconstruction was performed on cathode #2 using the Auto Slice and View technique on the FIB-SEM. Back scattered electron (BSE) micrographs were collected in automated mode by the FIB-SEM, and these were subsequently used for 3D reconstruction of the W crystal shape using Avizo visualization software (Avizo 9.3).

## 3. Results

### 3.1. Surface Morphology of Scandate Cathodes

**Figure 1:**
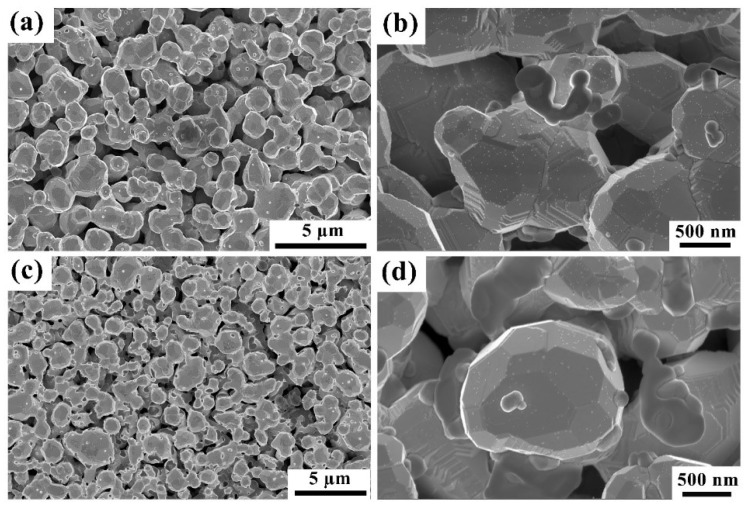
Surface morphology of two scandate cathodes. (**a**,**b**) are low- and high-magnification SEM images of the surface of cathode #1. (**c**,**d**) are low- and high- magnification SEM micrographs of cathode #2. All micrographs were obtained in secondary electron imaging mode.

Images of the surfaces of two scandate cathodes are presented in Figure 1. It is seen in Figure 1a,c that the W particle size is ~1 µm and the pores are open, i.e., not obstructed by impregnate material. Two types of particles, each with a size on the order of 100 nm, are distributed across the surfaces of W grains: Sc_2_O_3_ and BaAl_2_O_4_, as determined from EDS point analysis of composition. Complementary and more detailed analysis is also discussed in a related article [37]. Additionally, a critical observation, apparent in Figure 1b,d, is the highly faceted surface morphology of W grains in both cathodes. Based on Wulff analysis and as discussed in a separate publication [37], these facets correspond to the {100}, {110} and {112} crystallographic planes of W.

The nanoscale particles with diameter on the order of 10 nm, which decorate W facets in a fine dispersion, are assumed to be either Ba or BaO, based on results reported in the literature [37,38]. It appears, however, that numerous Ba/BaO particles were distributed over the W surfaces for cathode #1, while relatively fewer particles were observed on the surface of cathode #2, as shown in Figure 1b,d. As discussed in a previous paper [37], these Ba/BaO particles may result from transformation of a metallic Ba monolayer that exists at high temperature but is unstable during cooling after testing; this transformation may occur during cathode cooling after operation, creating the nanoscale particles observed here, and these particles would likely oxidize upon exposure to oxygen. Considering the emission test times, it may be reasonable to observe relatively lower areal density of Ba/BaO particles on the W facets of cathode #2, which had undergone emission testing for 9,840 h. This is more than three times the duration of testing for cathode #1, which could have resulted in lower (sub-monolayer) Ba coverage on the W surfaces of cathode #2 due to Ba loss during operation, leading to lower density of the Ba/BaO particles.

### 3.2. Elemental Analysis of Phases in Cross-Section Samples

**Figure 2:**
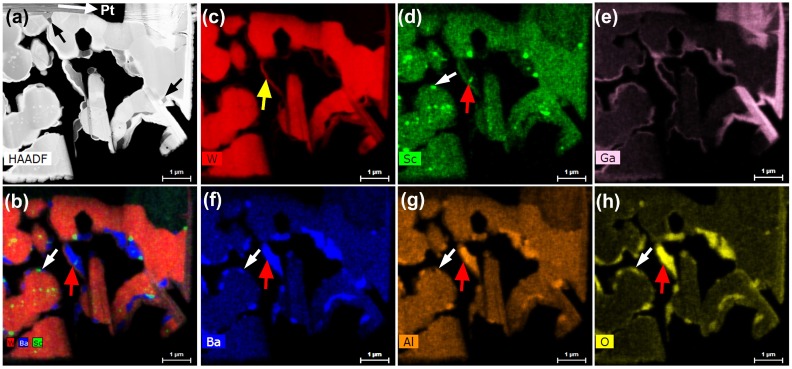
EDS elemental mapping of a cross-section TEM lamella from scandate cathode #1. (**a**) Low-magnification HAADF image of the electron-transparent sample region; (**b**) composite elemental map showing the distribution of W (red), Ba (blue) and Sc (green) in the TEM lamella; (**c**–**h**) elemental distribution maps for W, Sc, Ga, Ba, Al and O.

High-resolution elemental mapping was performed on a TEM lamella extracted from cathode #1, using the multi-spectrometer EDS system of the FEI Talos F200X TEM. These results are presented in Figure 2, where the images and elemental maps show a cross-section of the cathode that was created during preparation of the TEM lamella. This specimen therefore reveals the structure and composition of the near-surface cathode region, including what is inside the W particles as well as what is on their surfaces and in the pores. The high-angle annular dark field (HAADF) image in Figure 2a shows the equiaxed shape of W particles, with flat edges corresponding to different surface facets of a given particle. Pt was deposited on the top cathode surface for protection during subsequent ion milling, as indicated by the white arrow in this image. Figure 2b displays a composite map of composition across the lamella, with different colors corresponding to the major elements of interest, and Figure 2c–h display elemental maps for W, Sc, Ga, Ba, Al and O, respectively. Comparing Figure 2d,h, specifically the area marked by the white arrow, it is seen that some compounds attached to the edges of tungsten particles show consistent Sc and O signals during EDS mapping, while certain other Sc-containing compounds located inside tungsten particles (e.g., the row of 4 particles inside the W grain, below the white arrow in Figure 2d,h) do not exhibit a noticeable corresponding O signal. In addition to the Sc-containing particles, it was observed in the cross-section maps of Figure 2f–h that other particles exhibit highly correlated signals of Ba, Al and O. These particles are interpreted to have a mixed Ba–Al oxide phase. The majority of these Ba–Al oxides exhibit only Ba, Al and O signals, e.g., as indicated by the black arrows in Figure 2a. However, several of the Ba–Al oxides also exhibit simultaneous Sc and O signals. In these cases, the Sc–O appears to be entrapped within the Ba–Al oxides, as indicated by red arrows in Figure 2b, as well as Figure 2d,f,g. Note that the Sc–O particles are distinct from the Ba–Al oxide particles. In addition, a number of Ba–Al oxide particles are observed to be immediately adjacent to other particles that show Sc and O signals, as marked by white arrows in Figure 2b, as well as Figure 2d,f,g. It is noted that a shell of W encases some of the particles seen in the cross-section lift-out specimen, for instance as indicated by the yellow arrow in Figure 2c, but this is attributed to redeposition of sputtered W from the ion-milling process during fabrication of the TEM lamella. This is also consistent with the Ga map in Figure 2e, since Ga is present in the redeposited layers.

**Figure 3:**
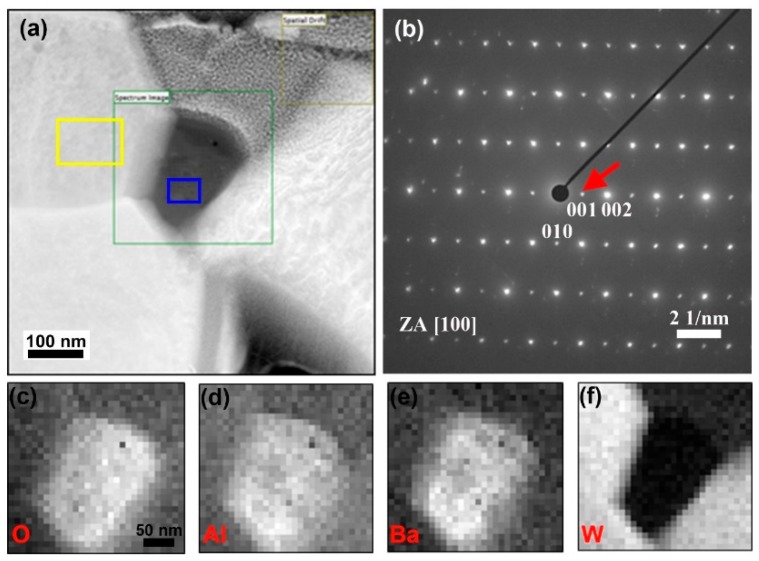
Structural and elemental analysis of a Ba–Al–O particle at the cathode surface. (**a**) HAADF image, with green square showing the area for EELS mapping, as well as blue and yellow boxes showing sites for EDS analysis of the Ba–Al–O particle and neighboring W grain, respectively; (**b**) selected area diffraction pattern acquired from the Ba–Al–O particle; (**c**–**f**) EELS elemental maps of O, Al, Ba and W; these four maps share the same scale bar.

The composition of a Ba–Al oxide particle near the cathode surface (visible as a dark particle at the upper left of Figure 2b), was investigated using EDS coupled with EELS. The results are presented in Figure 3. The HAADF image in Figure 3a shows the Ba–Al oxide particle located between three W particles and a Pt cap layer (from the FIB lift-out process). The EDS measurement of composition was taken from the region marked by the blue box in Figure 3a, and indicated the following composition: Ba (12.2 at.%), Al (20.7 at.%), O (63.4 at.%), W (0.5 at.%), and C (3.2 at.%). No Sc signal was detected here, i.e., no measurable peak was seen at 4.09 keV on the EDS spectrum acquired in the region outlined by the green box that encompasses the oxide particle. This measured composition provides a reasonable match to the compound BaAl_2_O_4_, albeit with excess O. Moreover, as shown in Figure 3b, a selected area electron diffraction pattern was obtained from the oxide particle, and it matched the known hexagonal crystal structure of BaAl_2_O_4_. Indexing of this diffraction pattern indicated that it corresponds to a [100] zone axis orientation, and it matches the theoretical pattern expected for BaAl_2_O_4_ in this orientation. A separate EDS measurement from a neighboring grain, in the region marked by the yellow box of Figure 3a, yielded a composition of W (78.5 at.%), Ba (0.1 at.%), Al (0.8 at.%), O (7.3 at.%) and C (13.3 at.%), confirming that the neighboring grain is W. The measured oxygen content of ~7 at.% and carbon content of ~13 at.% in the W area are assumed to arise from surface contamination, which is typical in such samples; it is noted that this would imply the EDS measurement in the blue box region more closely matches the expected stoichiometric composition of BaAl_2_O_4_. Figure 3c–f present the elemental maps acquired by EELS in the region outlined by the green box in Figure 3a. These maps indicate highly correlated signals of O, Al and Ba (but with an absence of W), in agreement with the EDS analysis.

**Figure 4:**
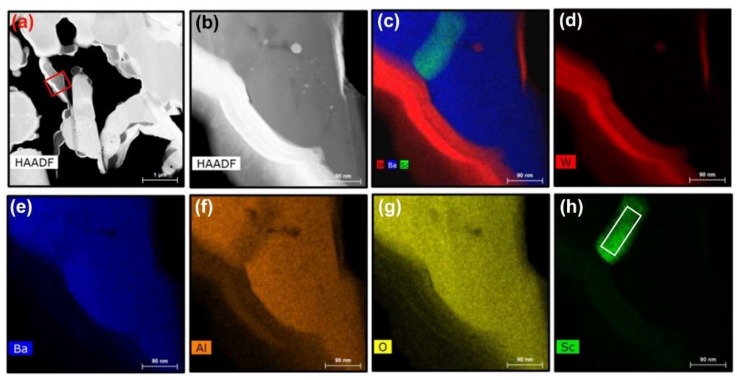
EDS elemental analysis of a Ba-Al oxide particle that also exhibits an encased Sc signal. (**a**) Low-magnification HAADF image of near-surface region of the cathode sample; (**b**) high- magnification HAADF image showing the area scanned for EDS mapping, which corresponds to the red box in image (**a**); (**c**) composite elemental map showing distributions of W, Ba and Sc; (**d**–**h**) individual elemental maps of W, Ba, Al, O and Sc, where the red box in (**h**) outlines the region of EDS quantitative measurement.

To explore the relationship between Ba–Al oxide and embedded Sc-oxide, the region denoted by red arrows in Figure 2 was studied in more detail and these results are presented in Figure 4. The HAADF images, where Figure 4b corresponds to the red box in Figure 4a, shows the morphology of the Ba–Al oxide region, where it is seen that Sc-oxide is surrounded by Ba–Al oxide. Figure 4c–h are composite and elemental EDS maps showing the distribution of W, Ba, Al, O and Sc. It is noted that the Sc signal is distinct and confined to one particle, as seen in Figure 4h. EDS measurement of composition in the region outlined by the red box in Figure 4h indicates the following: Ba (12.6 at.%), Al (20.5 at.%), O (56.3 at.%), Sc (10 at.%), W (0.5 at.%), and Ga (0.2 at.%). This suggests that the measured area includes both BaAl_2_O_4_ and Sc_2_O_3_, which likely exist as distinct but overlapping oxide particles.

**Figure 5:**
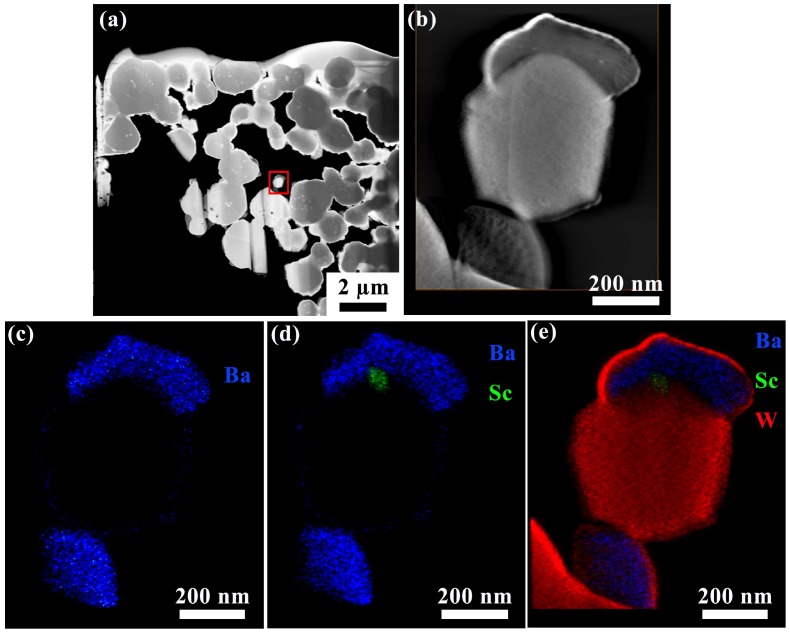
(**a**) HAADF image of near-surface cathode region where structure and elemental distribution were investigated by tomography. (**b**–**e**) HAADF image and EDS elemental maps, shown as 2D slices from the 3D tomogram, of a Ba–Al oxide particle that encases a smaller Sc-containing particle and sits on a W grain. Sc is distinct from both the W particle and the Ba–Al oxide.

In order to further investigate the relation between BaAl_2_O_4_ and Sc_2_O_3_, including possible physical mixing or chemical interactions between the oxides, a small W particle with attached oxides was selected for detailed visualization via a 3D EDS tomogram; this region is indicated by the red box in Figure 5a. It is noted that this experiment was conducted with a separate TEM lamella than the one imaged in Figure 1, Figure 2, Figure 3 and Figure 4, but both lamellae were cut from cathode #1. The higher magnification image in Figure 5b shows a BaAl_2_O_4_ particle atop the larger W particle. 2D slices from the 3D EDS tomogram are presented in Figure 5c–e, showing progressively more elements in each EDS map. This figure sequence conveys the relative distribution of Ba, Sc and W, and confirms that Sc is distinct from both the W particle and the BaAl_2_O_4_ particle that encases it, i.e., Sc does not appear to be physically or chemically mixed with W or BaAl_2_O_4_. Video Supplementary Video S1 shows the 3D tomogram in imaging mode and in EDS mapping mode, and also rotated about the vertical axis, is available in the online Supplemental Files. The EDS composite elemental mapping in this 3D tomography movie corroborates the observations described above and, along with the observations portrayed in Figure 1, Figure 2, Figure 3 and Figure 4, indicates that Sc exists in distinct Sc_2_O_3_ oxide particles that decorate the surfaces of W grains.

**Figure 6:**
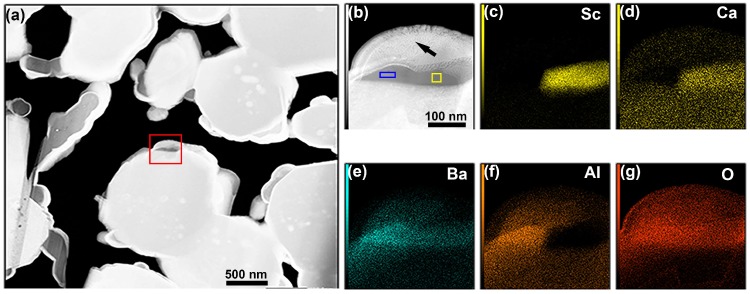
High spatial resolution EDS elemental mapping of Ba–Al oxide and adjacent Sc-containing particle. (**a**) Low-magnification HAADF image; (**b**) high-magnification HAADF image of area mapped by EDS, corresponding to red box in image (**a**), blue and yellow boxes denote locations of EDS point analysis; (**c**–**g**) elemental maps of Sc, Ca, Ba, Al and O; (**b**–**g**) share the same scale bar.

The Ba–Al oxide particle marked by the leftmost white arrow of Figure 2 was characterized at high-magnification, since it was adjacent to a Sc-oxide particle and offered an opportune location for more detailed study of these distinct but adjacent oxides. Figure 6 presents high spatial resolution images and elemental maps of these particles; as seen by comparison of Figure 2a and Figure 6a, the TEM lamella has been imaged from the opposite orientation in Figure 6, i.e., the features are reversed left-to-right. The HAADF image of Figure 6b, corresponding to the red box in Figure 6a, shows a wide darker gray region that indicates lower average atomic number and therefore suggests the presence of oxide particle(s). The elemental maps of Figure 6c–g agree with other observations of distinct BaAl_2_O_4_ and Sc_2_O_3_ particles and, moreover, they show a sharp boundary between Sc and Al. These high spatial resolution EDS maps also revealed a Ca signal, albeit a weak one. Ca may be expected to exist in the tested cathode, as it is intentionally added as part of the impregnate mix during cathode fabrication; however, Ca is not typically detected at significant levels during EDS analysis. However, when it is detected in the near-surface region of an activated and tested scandate cathode, the Ca signal is usually coupled with a Sc signal, as is the case in Figure 6. This may be due to the presence of a mixed Sc–Ca-oxide at certain locations, or remnant CaO (from the impregnate material) situated above/below the Sc_2_O_3_; however, the low number of such observed particles precludes a definitive statement at this point.

Quantitative EDS analysis was performed on the Ba-containing and Sc-containing oxide particles in Figure 6, in the regions marked by blue and yellow boxes in Figure 6b, respectively. These results are presented in Table 1. The region marked by the blue box had a composition (at.%) of Ba (9.3), Al (18.4), O (67.3), W (0.7), C (4.3), which is similar to the result for the oxide particle shown in Figure 3. This region is therefore interpreted to be BaAl_2_O_4_, which agrees with the EDS elemental maps in Figure 6. The region marked by the yellow box had a composition (at.%) of Sc (29.8), Ca (5.2), Ba (3.0), Al (0.2), O (60.7), W (1.1). Again, this agrees with observations described above, except for the presence of Ca. Given the additional presence of a low but not insignificant amount of Ba in the yellow box region, it is proposed that these signals may arise from remnant amounts of CaO and BaO that were added in the impregnate mix. Therefore, the primary oxide particle in the yellow box region would be Sc_2_O_3_, along with low amounts of CaO and BaO.

**Figure 7:**
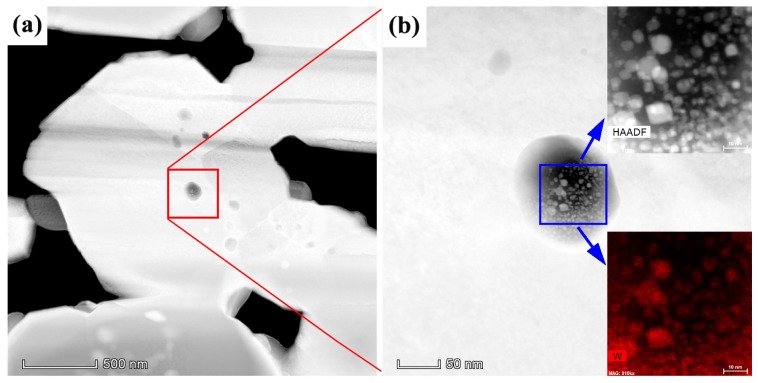
High spatial resolution characterization of a Sc-containing particle located in the interior region of a W grain. (**a**) Low-magnification HAADF image showing the near-surface region of the cathode; (**b**) zoomed HAADF micrograph corresponding to the red box in (**a**). Inset in (**b**) are a HAADF image and W elemental distribution map, revealing nanoscale W particles within the 100 nm Sc-containing particle.

In addition to the oxide particles that were observed at the surfaces and edges of W grains, the Sc-containing nanoparticles embedded in the interior regions of larger W particles were investigated using a combination of HAADF imaging and high spatial resolution EDS mapping, as shown in Figure 7. The red box in Figure 7a indicates the Sc particle of interest, with a corresponding magnified image in Figure 7b. The white spots dispersed throughout the Sc particle, as seen in the blue box of Figure 7b, appear to be spherical in shape, with a diameter in the range of 1–10 nm. The inset image and elemental map in Figure 7b reveal that the nanoscale particles dispersed throughout the larger (~100 nm) Sc-containing particle are W. A possible explanation for this curious result is that W may be etched during the L-S fabrication process, and W surfaces may become rough or pitted, allowing Sc_2_O_3_ to form in these pitted regions. This is discussed in more detail below.

### 3.3. Confirmation of Crystal Structure of W Grains

**Figure 8:**
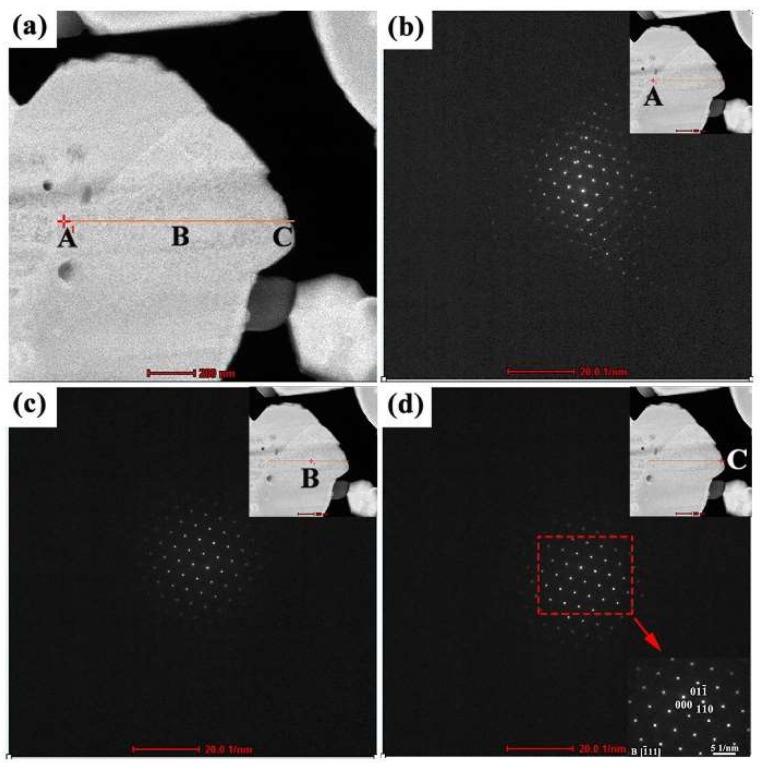
Crystal structure of the W matrix in scandate cathode #1, characterized using scanning nanobeam diffraction. (**a**) HAADF image showing the selected tungsten grain; (**b**–**d**) diffraction patterns corresponding to the sites of A, B and C as marked in (**a**). It is demonstrated that the W matrix has the BCC structure, which is inferred from diffraction patterns obtained at locations ranging from the middle of the W grain to its surface. The beam direction and zone axis for the grain orientation shown here are indexed as [−111].

α-W is the most common phase of tungsten, and it exists in the body centered cubic (BCC) crystal structure. The structure of the W particles that form the cathode body were studied in more detail here. It would be convenient if the structure of W in the cathode body were the same as ubiquitous α-W, but given the complex chemical reactions that take place during preparation and emission testing of a cathode, this may not be true, and therefore the crystal structure was characterized. Scanning nanobeam diffraction was performed on a TEM lamella extracted from cathode #1. In this diffraction experiment, the beam was shifted along a line-scan direction with a step size of 20 nm, from point A to point C in Figure 8a, in order to determine the crystal structure at regular intervals from the grain interior to its surface. Diffraction patterns at three points of this line scan are displayed in Figure 8b–d, which correspond to sites A, B, and C, respectively. In all cases, the diffraction pattern corresponds to a typical BCC structure oriented along a <111> zone axis. The diffraction patterns obtained at sites B and C are identical. The indexed diffraction pattern displayed in the lower right corner of Figure 8d is representative of all locations characterized in this investigation, confirming that the W grains exist in the BCC crystal structure. In other words, the W matrix has not undergone significant changes due to activation or emission testing. Note that Supplementary Video S2 portrays the scanning nanobeam diffraction experiment and includes the diffraction patterns recorded at each 20 nm step position.

### 3.4. 3D Tomographic Reconstruction

**Figure 9:**
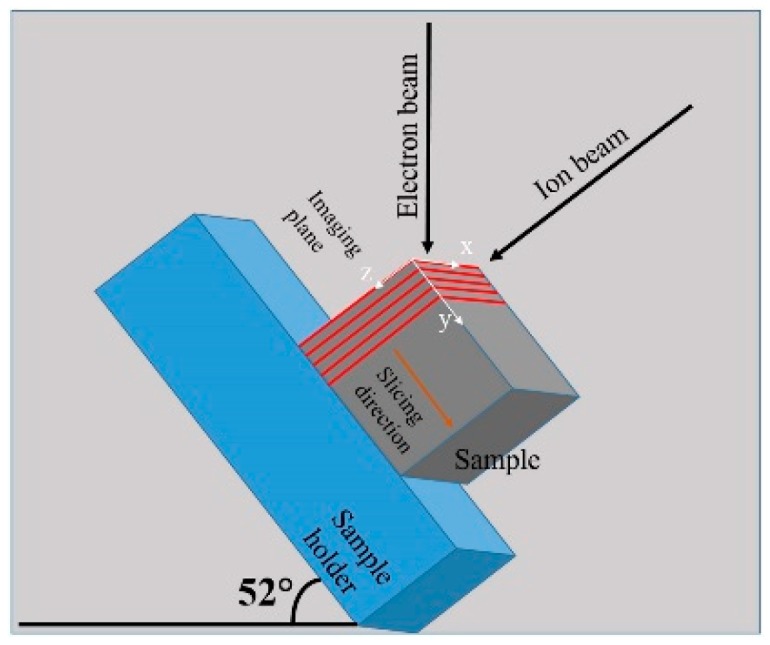
Schematic illustration of the sample, ion beam and electron beam for serial sectioning in the FIB-SEM, in order to perform 3D tomographic reconstruction.

Standard characterization techniques such as SEM, TEM and AFM typically reveal planar, projected or surface information, and therefore may not be ideal for revealing internal microstructure. As such, 3D reconstruction can provide insights into the microstructure of a complex 3D object that is not attainable with 2D characterization [39]. In the current study, a 3D tomogram was constructed for the first time on a scandate cathode, by employing the dual-beam FIB-SEM microscope. This technique involves layer-by-layer milling/removal of thin slices of the sample, using the ion beam, with SEM images recorded after each slice. 10 nm thick slices were milled sequentially, culminating in 137 backscattered electron (BSE) images stitched together to form a 3D tomogram. A schematic of the serial sectioning technique is presented in Figure 9. In order to perform FIB milling, the sample stage is tilted to 52° and the region of interest is situated at the eucentric height, which is where the sample region of interest will not change its vertical position during tilting (it is also where the ion beam and the electron beam converge). Prior to slicing, the sample was coated with a layer of Pt to protect the underlying region of interest. The *x*-*y* plane is perpendicular to the ion beam, and the *x*-*z* plane (parallel to the ion beam) is imaged after each slice. The red lines in Figure 9 represent the serial sectioning that is performed at regular intervals along the *y*-axis of the sample.

**Figure 10:**
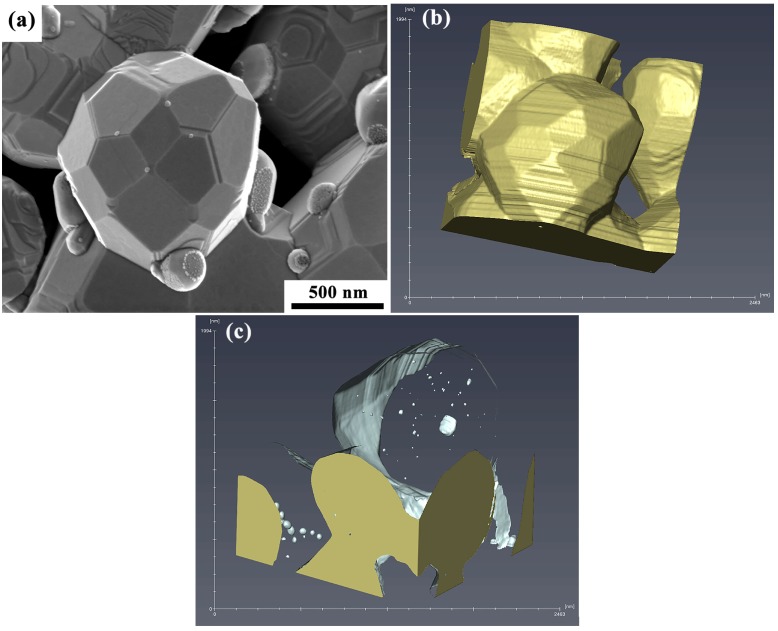
3D reconstruction of a W particle from scandate cathode #2, generated by FIB serial sectioning and imaging in the SEM. (**a**) Secondary electron SEM micrograph of the selected W particle, (**b**) reconstructed tomogram, and (**c**) spatial distribution of Sc/Sc_2_O_3_ inside the W particle. Note that the viewing direction of image (**c**) is rotated with respect to that of images (**a**,**b**).

The 3D reconstructed shape of a particular W particle was generated using Avizo software, and involved 137 BSE micrographs obtained after each step of automated serial sectioning. This W particle is shown in Figure 10a and exhibits surface facets. A noise reduction filter was applied to each micrograph prior to reconstruction. Each image was aligned with respect to a fiducial mark, resulting in the reconstructed tomogram displayed in Figure 10b. The SEM image of the selected W particle in Figure 10a and the 3D reconstruction of that particle in Figure 10b agree well with each other, and the surface facets are consistent and clear in both figures. The linear surface steps in Figure 10b result from the serial slicing process, specifically from slight misalignments between image positions after each slice, i.e., these surface steps are an artifact of reconstruction. Since BSE imaging was used to generate the tomogram, it was possible to optimize atomic number contrast for setting thresholds in the reconstruction. The metallic W regions exhibited consistent brightness levels, while the oxide particles were darker gray and clearly distinguishable from the main W particle. Leveraging this contrast, the (darker) oxide particles were differentiated, and Figure 10c reveals Sc/Sc_2_O_3_ particles trapped inside the larger W particle. As shown in Movie Supplementary Movie S3, where the tomogram of Figure 10c is rotated and viewed from multiple directions, the smaller oxide particles can be seen to lie inside the W particle. These observations are consistent with the results described earlier, namely that Sc/Sc_2_O_3_ particles are contained inside larger W grains.

**Figure 11:**
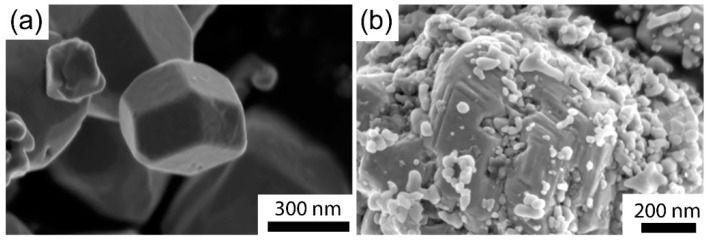
SEM micrographs of (**a**) tungsten powder and (**b**) scandia-doped tungsten powder. Both images were recorded in secondary electron imaging mode.

To further investigate the source of the Sc_2_O_3_ particles that were observed inside the W grains of the cathode pellet, the starting materials were characterized. This included pure W powder, as well as Sc_2_O_3_-doped W powder that had been processed from the same W powder using the L-S technique. Figure 11 presents SEM micrographs of each powder set, showing particle morphology and distribution. As seen in Figure 11a, the W particles have clean, flat surface facets prior to the L-S processing step. However, after the L-S stage, W surfaces are decorated with Sc_2_O_3_ particles and W exhibits microfacets at the intersections of neighboring facets. These microfacets may result from etching of W during L-S processing. Additionally, there are numerous cavities that appear to have been etched into the W grains, following by backfilling of these cavities with Sc_2_O_3_ particles. An example of this is shown in the central region of Figure 11b. Thus, the L-S processing step may allow Sc_2_O_3_ to penetrate some distance into W grains, which later form seemingly regular, faceted W grains during high-temperature activation and emission testing stages. In this way, the Sc_2_O_3_ particles may become trapped within W, resulting in the microstructural features that were described above.

## 4. Discussion

Since the primary materials difference between scandate cathodes and conventional impregnated tungsten cathodes is the addition of scandia to the W matrix, it is reasonable to assume that scandia (or scandium) is responsible for the significant improvement in thermionic emission and the low work function of scandate cathodes. However, the exact role of Sc/Sc_2_O_3_ in cathode processing and/or performance remains unclear. Uda et al. [40] suggested it is impossible for Sc_2_O_3_ to migrate from interior regions of the porous W matrix to the cathode surface during activation, due to the low diffusion coefficient (1.6 × 10^−18^ cm^2^/s at 1370 K). Therefore, in order for Sc to diffuse or migrate to the cathode surface within a reasonable timeframe, Sc_2_O_3_ would need to be transformed into metallic Sc, as proposed by Wang [41] and Liu [32]. However, the reaction(s) required to generate free Sc and the mechanism of Sc migration have not been experimentally verified, despite these research efforts. In this section, chemical reactions that may enable the creation of metallic Sc are discussed, as are Sc-containing compounds that may be relevant to the role of Sc in scandate cathodes.

Sc has a stronger affinity for O than does Ba, which means that Ba cannot reduce Sc_2_O_3_ to Sc directly [9]. In a cathode study by Yamamoto et al. [35], it was proposed that metallic Sc can be produced via a chemical reaction between Sc_2_W_3_O_12_ and Ba, as described in Equation (1), which was based on their work on (W–Sc_2_O_3_)-coated [42] and (W–Sc_2_W_3_O_12_)-coated impregnated cathodes [35].

Sc_2_W_3_O_12_ + 3Ba = 3BaWO_4_ + 2Sc (1)

In that reaction scenario, metallic Ba was created by impregnate materials reacting with W at pore walls, similar to the case for regular impregnated tungsten cathodes. Multi-step chemical reactions that generate Ba were described by Jones [18] as:

5BaO + 2Al_2_O_3_ = 3/2 Ba_3_Al_2_O_6_ + 1/2 BaAl_2_O_4_ (2)

2Ba_3_Al_2_O_6_ (s) + W (s) = BaWO_4_ (s) + 2BaAl_2_O_4_ (s) + 3Ba (g) (3)

According to these reactions, the production of free metallic Ba is accompanied by generation of the compound BaAl_2_O_4_. As demonstrated by Rittner [43], this compound can be regarded as an inert material, i.e. it is thermally stable and does not participate in thermionic electron emission from the cathode surface. Since Equations (2) and (3) take place at W pore walls, i.e., the surfaces of W grains, it is reasonable to observe BaAl_2_O_4_ particles at W surfaces and junctions in cathode cross-section samples, as was shown for example in Figure 3.

As described by Yamamoto [35], the compound Sc_2_W_3_O_12_, which can result from oxidation during heat treatment of W–Sc_2_O_3_, is the most likely source of metallic Sc and would be produced according to Equation (1) above. However, Sc_2_W_3_O_12_ is not observed in the cathode samples described in the current paper, and this may be due to complete reaction of the compound Sc_2_W_3_O_12_. By virtue of their nanoscale size and uniform distribution across W surfaces, the Sc_2_O_3_ nanoparticles fabricated during L-S processing in the current study may react more readily with W, and thereby enhance the formation of Sc_2_W_3_O_12_. If these mixed-oxide particles are also sufficiently small and uniformly distributed over W pore walls, this would facilitate the creation of free metallic Sc throughout the porous network.

Additionally, according to Slooten [44], the rate of Sc supply is significant only in the initial hours of cathode operation. After this transient stage, the generation of metallic Sc decreases to an undetectable level due to the formation of stable compounds such as Ba_3_Sc_4_O_9_ or Ba_2_ScAlO_5_, which inhibit electron emission. However, these phases were not observed during the characterization work in the current study. Future efforts will include a more detailed study of Sc-containing compounds in scandate cathodes.

The exact role of Sc in scandate cathode processing and performance remains elusive, although there is now an improved understanding of the various phases in the near-surface regions of scandate cathodes. Other studies in the scientific literature ascribe the enhanced emission capability and low work function of scandate cathodes to a Ba–Sc–O monolayer [29,45] or a relatively thick (~100 nm) Ba–Sc–O layer [7,8,32] that covers W grains at the cathode surface. However, as was described in a recent paper from the current authors’ group [37,46], no Ba–Sc–O layer with thickness on the order of 10–100 nm was observed, despite using several pertinent and complementary techniques in the characterization of multiple scandate cathodes that had yielded good emission test results. Finally, as suggested by Hasker [29] and Zhou [46], one possible function of Sc is to regulate the chemical potential of oxygen on the emitting surface of a scandate cathode, since the affinity of O for Sc is greater than it is for Ba.

## 5. Conclusions

Multiple scandate cathodes were prepared using the liquid-solid fabrication method and were subjected to CSD emission testing. Subsequently, they were characterized using multiple advanced microscopy techniques, including (S)TEM imaging, high spatial resolution EDS mapping, EELS analysis, scanning nanobeam diffraction, and 3D reconstruction/tomography. The experimental observations and discussion above are summarized with these concluding remarks:

- Most of the larger (~100 nm diameter) particles that are attached to the surfaces and edges of W grains are BaAl_2_O_4_, as revealed during characterization of scandate cathode cross-section samples. In some cases, BaAl_2_O_4_ particles are contiguous with Sc_2_O_3_ particles and/or envelop Sc_2_O_3_ particles.
- No evidence for chemical interaction or mixing between BaAl_2_O_4_ and Sc_2_O_3_ was observed, i.e., these oxide particles appear to remain distinct.
- Scanning nanobeam electron diffraction in the TEM showed that the crystal structure of W grains remains body-centered cubic in emission-tested scandate cathodes. The crystal structure and lattice constant of W particles have not changed as a result of the complex chemical reactions that occur during cathode fabrication, nor during activation or emission testing.
- 3D tomographic reconstruction revealed the interior microstructure of W grains in a scandate cathode. Interestingly, nanoscale Sc/Sc_2_O_3_ particles tend to cluster or align in certain regions of a given W grain, rather than being distributed uniformly throughout the W grain.

## Figures and Tables

**Table 1:** Quantified measurement of particle composition, obtained from EDS point analysis of the regions marked in Figure 6.

EDS Site	Measured Composition (at.%)	Compound(s) Present
Blue box	Ba (9.3), Al (18.4), O (67.3), W (0.7), C (4.3)	BaAl_2_O_4_
Yellow box	Sc (29.8), Ca (5.2), Ba (3.0), Al (0.2), O (60.7), W (1.1)	Sc_2_O_3_ + minor levels of impregnate materials (CaO and BaO)

## Supplementary Materials

The following are available online at https://www.mdpi.com/1996-1944/12/4/636/s1, Video S1: 3D tomogram in Figure 5, Video S2: Scanning nanobeam diffraction in Figure 8, Video S3: 3D reconstruction in Figure 10.
